# The future of Italian phase I trials regulation: lessons from a nationwide survey

**DOI:** 10.3389/fmed.2025.1709108

**Published:** 2025-11-12

**Authors:** Celeste Cagnazzo, Linda Penolazzi, Stefano Stabile, Veronica Franchina, Irene Federici, Francesca Mannozzi, Alessandra Ferrari, Marta Betti, Sara Testoni

**Affiliations:** 1 Ospedale Infantile Regina Margherita – AOU Città della Salute e della Scienza di Torino, Turin, Italy; 2 ASST Grande Ospedale Metropolitano Niguarda, Milan, Italy; 3 A.O. Papardo, Messina, Italy; 4 AOU delle Marche, Ancona, Italy; 5 IRCCS Istituto Romagnolo per lo Studio dei Tumori (IRST) “Dino Amadori,” Meldola, Italy; 6 Fondazione IRCCS Policlinico San Matteo, Pavia, Italy; 7 Azienda Ospedaliero-Universitaria “SS Antonio e Biagio e C. Arrigo,” Alessandria, Italy

**Keywords:** quality, fase I, clinical research, autocertification, requirements

## Abstract

**Background:**

Phase I trials are critical for drug development and require rigorous oversight. In Italy, AIFA Determination 809/2015 introduced mandatory standards and a self-certification model, nearly a decade ago. Its sustainability and impact now warrant reassessment.

**Methods:**

A nationwide cross-sectional survey (March–April 2024) was conducted among professionals involved in phase I trials. A 19-item questionnaire explored institutional characteristics, certification processes, quality structures and perceptions of the Determination. Descriptive analyses were performed.

**Results:**

Sixty-two professionals responded, mainly Data Managers/Clinical Research Coordinators and Quality Assurance officers. Most centers conducted both industry and non-profit studies. Certification of both clinical units and laboratories was common, but timelines varied widely and preparation was resource-intensive. Over half of inspected centers reported major or critical deviations and voluntary suspensions of certification were not infrequent. Clinical Trial Quality Teams were established in most centers, though key roles were often outsourced. Respondents identified procedure drafting and staff training as the most burdensome requirements and considered parts of the Determination outdated, particularly regarding team composition and personnel qualifications. Comparative references with other European frameworks (e.g., Spain and the United Kingdom) highlight differences in implementation models and timelines.

**Conclusion:**

While AIFA Determination 809/2015 has strengthened safety and quality culture, it imposes significant operational burdens, especially on academic institutions. Targeted revision appears necessary to maintain high standards while improving sustainability and competitiveness of Italian phase I research.

## Introduction

### State of the art

Phase I clinical trials represent the first and most critical step in the clinical development pathway, bridging preclinical findings into human application. Designed to assess safety, tolerability, pharmacokinetics (PK), pharmacodynamics (PD) and to identify the Maximum Tolerated Dose (MTD), these studies construct the scientific and ethical foundation for subsequent drug development stages (1, 2). While traditionally conducted in small cohorts of healthy volunteers, phase I oncology trials involve patients with advanced or refractory disease, for whom participation may represent the only remaining therapeutic opportunity, where the potential clinical benefits may outweigh the possible risks associated with first-in-human (FIH) exposure (1).

Given their exploratory nature and elevated risk profile, phase I trials carry substantial ethical and scientific responsibilities. Historical incidents such as the TGN1412 cytokine storm (3) and the BIA 10-2474 neurotoxicity event (4), have dramatically shown the potential risks of early phase trials and underscored the importance of rigorous safety oversight. Consequently, international regulatory frameworks, anchored in the Declaration of Helsinki and the Good Clinical Practice (GCP) principles (5), have progressively evolved to mitigate risk and enhance participant protection (1).

In Italy, a key regulatory milestone was achieved with the introduction of AIFA Determination No. 809/2015 (6), fully effective from July 2016. This determination established the minimum mandatory standards requirements for clinical units and laboratories conducting phase I studies, including structural requirements, personnel qualifications, emergency procedures, documentation practices and the implementation of comprehensive Quality Management Systems (QMS) with dedicated operating procedures (SOPs) in order to meet increasingly stringent quality and compliance standards (1, 7–9). A notable innovation was the introduction of a self-certification model (10) whereby institutions autonomously declare compliance with national requirements (4), shifting the focus from sponsor-led feasibility to a nationally regulated model with a centralized registry of authorized phase I centers maintained by the Italian regulatory agency (AIFA) (2, 11).

This shift marked a paradigm change in the Italian regulatory landscape intended to foster transparency, standardization and reliability across institutions. The 809/2015 Determination also promoted the development and adoption of internal Clinical Trial Quality Teams (CTQTs), multidisciplinary units responsible for ensuring regulatory and quality compliance, particularly in academic study settings (2, 9). However, while the AIFA Determination 809/2015 has improved safety standards and harmonized processes for phase I trials in Italy, its rigid and resource-intensive implementation continues to place a significant disproportionate operational burden on clinical research centers, especially for public hospitals and academic institutions (2), where the need to meet highly specialized and costly infrastructural, procedural and personnel requirements often exceeds available institutional resources (11). Comparative analyses across European countries have further contextualized the Italian regulatory landscape. Both the British MHRA Phase I Accreditation Scheme and the Italian AIFA Determination 809/2015 share a quality-risk-management foundation but differ substantially in implementation. The United Kingdom applies a voluntary accreditation system with periodic inspections and certification renewal, while Italy relies on a mandatory national self-certification framework overseen by the regulatory authority. These distinct approaches highlight how different regulatory philosophies can shape feasibility, sustainability and competitiveness in early phase clinical research (8).

In contrast, Spain rapidly adopted the new EU Regulation No. 536/2014 (12) into national practice, adopting centralized trial application and simplified authorization procedures. This model has demonstrated increased competitiveness in early phase research, through simplified, less bureaucratic processes (2). Italy’s slower adaptation and continued reliance on a complex and costly national self-certification process, may be contributing to its lag in international trial participation. Despite a strong scientific reputation, Italy currently ranks last among the five largest European countries in terms of the number of registered phase I trials (2).

In light of these dynamics, this study aimed to capture the perspectives of professionals directly engaged in the management and conduction of phase I trials in Italy, including investigators, Data Managers (DM), Clinical Research Coordinators (CRC), Quality Assurance (QA) specialists, pharmacists and laboratory staff. Specifically, we sought to assess whether AIFA Determination 809/2015 remains fit for purpose and to explore any perceived need for revision. Through this national survey, we aim to inform potential improvements to Italy’s regulatory framework and support more agile and efficient early phase clinical research, particularly within the academic and non-profit sectors.

## Materials and methods

### Study design and objectives

This study was designed as a nationwide, cross-sectional survey aimed at capturing the experiences and perspectives of professionals directly involved in the planning, conduct and oversight of phase I clinical trials in Italy. The primary objective was to evaluate the perceived strengths, weaknesses and implementation challenges of AIFA Determination No. 809/2015, with a focus on its applicability to both profit and non-profit research settings.

### Survey development and pre-testing

The questionnaire was developed by a multidisciplinary working group composed of CRCs, QA experts and academic researchers with extensive experience in early phase oncology trials. Survey items were informed by regulatory frameworks (AIFA Determination 809/2015, EU Regulation 536/2014) and literature on operational barriers in early phase research. The survey instrument was piloted with a small group of clinical professionals (*n* = 10 GIDM’s members) to ensure clarity, content validity and usability. Based on feedback, minor revisions were made to the wording and order of items prior to deployment.

### Survey structure and content

**FIGURE 1:**
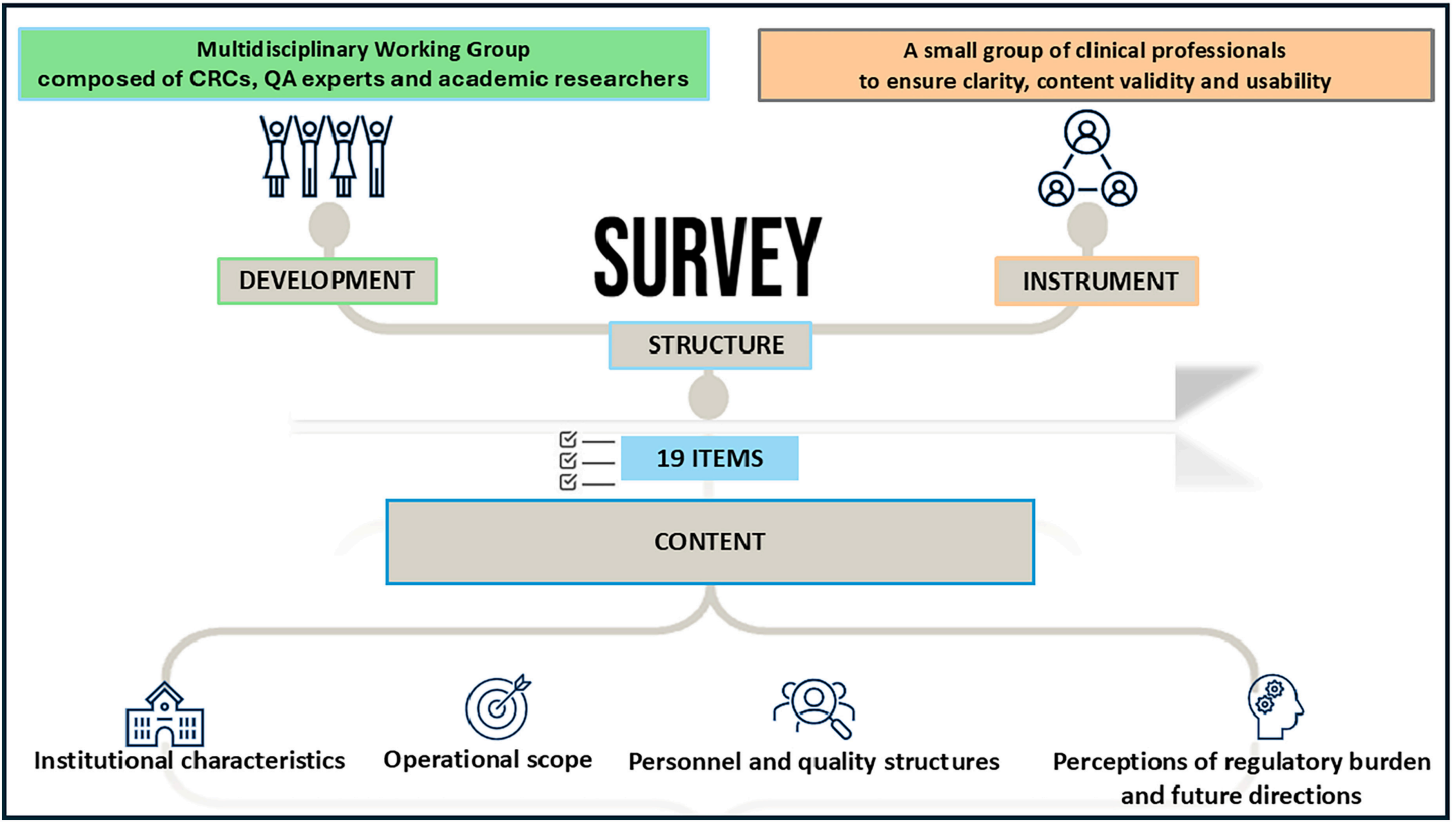
Survey structure and elements.

The final questionnaire included 19 items and was administered in Italian using Google Forms^®^. It featured a combination of multiple-choice questions, Likert-scale ratings (1–10) and short open-text responses. The content covered four domains (Figure 1):

1. *Institutional characteristics* (e.g., type of research center, year of self-certification, research setting)
2. *Operational scope* (e.g., volume of phase I studies, involvement in non-profit trials)
3. *Personnel and quality structures* (e.g., presence of certified monitors, CTQT teams, SOPs)
4. *Perceptions of regulatory burden and future directions* (e.g., feasibility of extending Determination 809/2015 to other trial phases; perceived need for regulatory update)

A copy of the full questionnaire is available in Supplementary material.

### Survey dissemination

The questionnaire was disseminated through multiple channels, including the GIDMcrc professional network, institutional mailing lists and social media groups for clinical research professionals.

### Sampling and participant recruitment

The target population included clinical research professionals affiliated with Italian centers conducting or aspiring to conduct phase I trials. Eligible participants included investigators, Clinical Research Coordinators (CRCs), Data Managers (DMs), Quality Assurance (QA) staff, pharmacists and laboratory managers. The survey was disseminated through the GIDMcrc network (Italian Clinical Research Coordinators and Data Managers Group), mailing lists and professional social media platforms.

Given the nature of the survey and the dissemination channels, an *a priori* sample was not defined. Participation was voluntary, anonymous and did not involve any financial incentive. Each participant provided implied consent by completing and submitting the survey. Duplicate responses from the same center were excluded based on institutional identifiers.

### Data collection and timeframe

Data collection occurred between March and April 2024. The average time required to complete the survey was approximately 15 min. A reminder email was sent to the target network 1 week after launch to increase response rates.

### Data analysis

Descriptive statistics were used to summarize responses. Categorical variables were reported as absolute frequencies and percentages. Ordinal variables (e.g., Likert-scale scores), treated as continuous for descriptive purposes, were described using medians and the frequency of each response option was categorized as “insufficient” for responses equal to or < 5 and sufficient for responses equal to or > 6. This threshold was chosen in line with conventional interpretation of Likert scales in similar surveys. To evaluate the responses, a cross-tabulation analysis was used, allowing for a detailed examination of the relationships between different variables. No imputation was performed for missing data. Analyses were conducted using Microsoft Excel^®^ software.

### Ethical considerations

The study did not involve human subjects, biological specimens or identifiable personal data. As such, formal ethical review was not required under Italian regulations for anonymous survey-based research. The study was conducted in accordance with national data protection laws and the Declaration of Helsinki.

## Results

A total of 62 professionals completed the survey and all responses were considered valid for the analysis based on the survey’s characteristics and predefined criteria. The most frequently represented roles were Data Manager/Clinical Research Coordinator (*n* = 31/62, 50.0%), followed by Quality Assurance Officers (*n* = 18/62, 29.0%). Other roles included clinician (*n* = 3/62, 4.8%), pharmacist (*n* = 3/62, 4.8%), research nurse (*n* = 2/62, 3.2%). Single entries for positions such as biologist or administrative officer.

A majority of respondents (67.8%, *n* = 42/62) reported involvement in both industry-sponsored and non-profit trials, whereas 29.0% (*n* = 18/62) conducted only industry-sponsored studies. Only 3.2% (*n* = 2/62) worked exclusively on non-profit trials.

When asked about the medical area of reference of their institution’s phase I unit, responses were highly heterogeneous.

Table 1 summarized the respondents’ characteristics.

**TABLE 1:** Characteristics of respondents.

Professional role	Count	Percentage
Data manager/clinical research coordinator	31	50.0
Quality assurance officer	18	29.0
Clinician	3	4.8
Pharmacist	3	4.8
Research nurse	2	3.2
Phase I program lead	1	1.6
Biologist	1	1.6
Administrative officer	1	1.6
Hospital medical director	1	1.6
Head of phase I laboratory	1	1.6
Total	62	100
**Sponsor type**	**Count**	**Percentage**
Both (profit and non-profit)	42	67.8
For-profit	18	29.0
Non-profit	2	3.2
Total	62	100
**Medical area**	**Count**	**Percentage**
Dedicated phase I unit for all therapeutic areas	19	30.6
Oncology	13	21.0
Oncology, hematology	11	17.7
Hematology	7	11.3
Oncology, gastroenterology	2	3.2
Others	10	16.0
Total	62	100

Percentages are rounded to one decimal place.

The most frequently reported were phase I units dedicated to all therapeutic areas (30.6%, *n* = 19/62), oncology (21.0%, *n* = 13/62), oncology and hematology combined (17.7%, *n* = 11/62) and hematology alone (11.3%, *n* = 7/62). Other medical areas, such as pediatrics, neurosciences, transplantation, gastroenterology, neonatal intensive care and medical genetics, were reported less frequently. Regarding institutional self-certification under AIFA Determination 809/2015, most respondents 71.0% (*n* = 44/62) reported that their institution had certified both a clinical unit and at least one laboratory (Table 2). Certification of clinical unit alone was reported by 27.4% (*n* = 17/62), while certification of the laboratory alone was uncommon (1.6%, *n* = 1/62). Among certified laboratories (Table 2), clinical analysis laboratories were the most frequently indicated (33.9%, *n* = 21/62), followed by microbiology and pathology laboratories, often in combination with each other or with preclinical facilities. A smaller number of respondents reported simultaneous certification of multiple laboratories, including immunohematology, transfusion, preclinical or biomarker assay facilities. With regard to certification history (Table 3), the timing of self-certification varied. A significant proportion of centers achieved compliance soon after the Determination came into force, with 32.3% (*n* = 20/62) reporting certification in 2016, the year the regulation became effective. Further peaks were observed in 2017 (21.0%, *n* = 13/62) and 2019 (9.7%, *n* = 6/62), with sporadic certifications continuing through to 2024.

**TABLE 2:** Details about certifications.

Certified units	Count	Percentage
Both (clinical units and laboratories)	44	71.0
Clinical unit (s)	17	27.4
Laboratory/ies	1	1.6
Total	62	100
**Laboratory type**	**Count**	**Percentage**
Clinical analysis laboratory	21	33.9
Unknown	15	24.2
Clinical analysis laboratory, microbiology laboratory	9	14.5
Clinical analysis laboratory, pathology laboratory, microbiology laboratory	8	12.9
Clinical analysis laboratory, pathology laboratory	4	6.5
Clinical analysis laboratory, pathology laboratory, microbiology laboratory, all	1	1.6
Clinical analysis laboratory, pathology laboratory, pre-clinical laboratory	1	1.6
Clinical trials laboratory for biomarker assays	1	1.6
Hematology laboratory	1	1.6
Clinical analysis laboratory, pathology laboratory, microbiology laboratory, immunohematology and transfusion laboratory	1	1.6
Total	62	100

Percentages are rounded to one decimal place. Clarification: “Unknown” indicates that the information was not provided by the respondent.

**TABLE 3:** Year of certification of phase I units.

Year of certification	Count	Percentage
1997	1	1.6
2008	1	1.6
2015	1	1.6
2016	20	32.3
2017	13	21.0
2018	5	8.1
2019	6	9.7
2020	2	3.2
2021	2	3.2
2022	5	8.1
2023	4	6.5
2024	2	3.2
Total	62	100

Percentages are rounded to one decimal place.

The duration of the preparatory phase for self-certification ranged from 1 to 50 months (Table 4), with 12 months being the most frequently cited timeframe (33.9%, *n* = 21/62), indicating the substantial organizational effort required by most respondents. Concerning the study population, the majority (74.2%, *n* = 46/62) stated that certification applied to studies involving patients only, while 25.8% (*n* = 16/62) included both patients and healthy volunteers (Table 5).

**TABLE 4:** Duration of preparation for self-certification (including missing data).

Preparation duration (months)	Count	Percentage
3	2	3.2
4	2	3.2
5	4	6.5
6	7	11.3
7	1	1.6
8	2	3.2
10	4	6.5
12	21	33.9
13	1	1.6
17	1	1.6
18	2	3.2
20	1	1.6
24	8	12.9
36	3	4.8
50	1	1.6
Unknown	2	3.2
Total	62	99.9

Percentages are calculated on the total of 62 responses and are rounded to one decimal place. Slight discrepancies may be due to rounding. Clarification: “Unknown” indicates that the information was not provided by the respondent.

**TABLE 5:** Other details about certified institutions.

Study population	Count	Percentage
Patients	46	74.2
Both (patients and healthy volunteers)	16	25.8
Total	62	100
**Annual No. of phase I studies**	**Count**	**Percentage**
< 2	15	24.2
2–5	21	33.9
6–10	13	21.0
> 10	13	21.0
Total	62	100
**AIFA inspection**	**Count**	**Percentage**
Yes, on-site	41	66.1
No	18	29.0
Yes, remote	3	4.8
Total	62	100

Percentages are rounded to one decimal.

When asked about the annual number of phase I trials activated in their unit (Table 5), 33.9% (*n* = 21/62) reported 2–5 phase I studies, while 24.2% (*n* = 15/62) activated fewer than two. Equal proportions (21.0%, *n* = 13/62 each) reported activating 6–10 and more than 10 studies per year, respectively.

Overall, 71.0% (*n* = 44/62) of respondents indicated that their centers had undergone an AIFA inspection (Table 5), either on site (66.1%) or remotely (4.8%). Among these, 56.8% (*n* = 25/44) stated that major or critical deviations had been identified (Table 6), while 40.9% (*n* = 18/44) reported no major or critical deviations; one respondent did not provide an answer. Notably, 21.0% (*n* = 13/62) of all participants reported that their clinical unit or laboratory had voluntarily suspended its certification (notifying AIFA) at least once, suggesting either critical non-conformities or operational limitations in maintaining compliance. The remaining 79.0% (*n* = 49/62) had never initiated a self-suspension.

**TABLE 6:** Severity of deviations identified during inspections.

Deviation severity	Count	Percentage
Yes	25	58.1
No	18	41.9
Total	43	100

Denominator restricted to inspected units with a valid response (*n* = 43); 1 missing among inspected (information was not provided by the respondent). Percentages are rounded to one decimal place.

**FIGURE 2:**
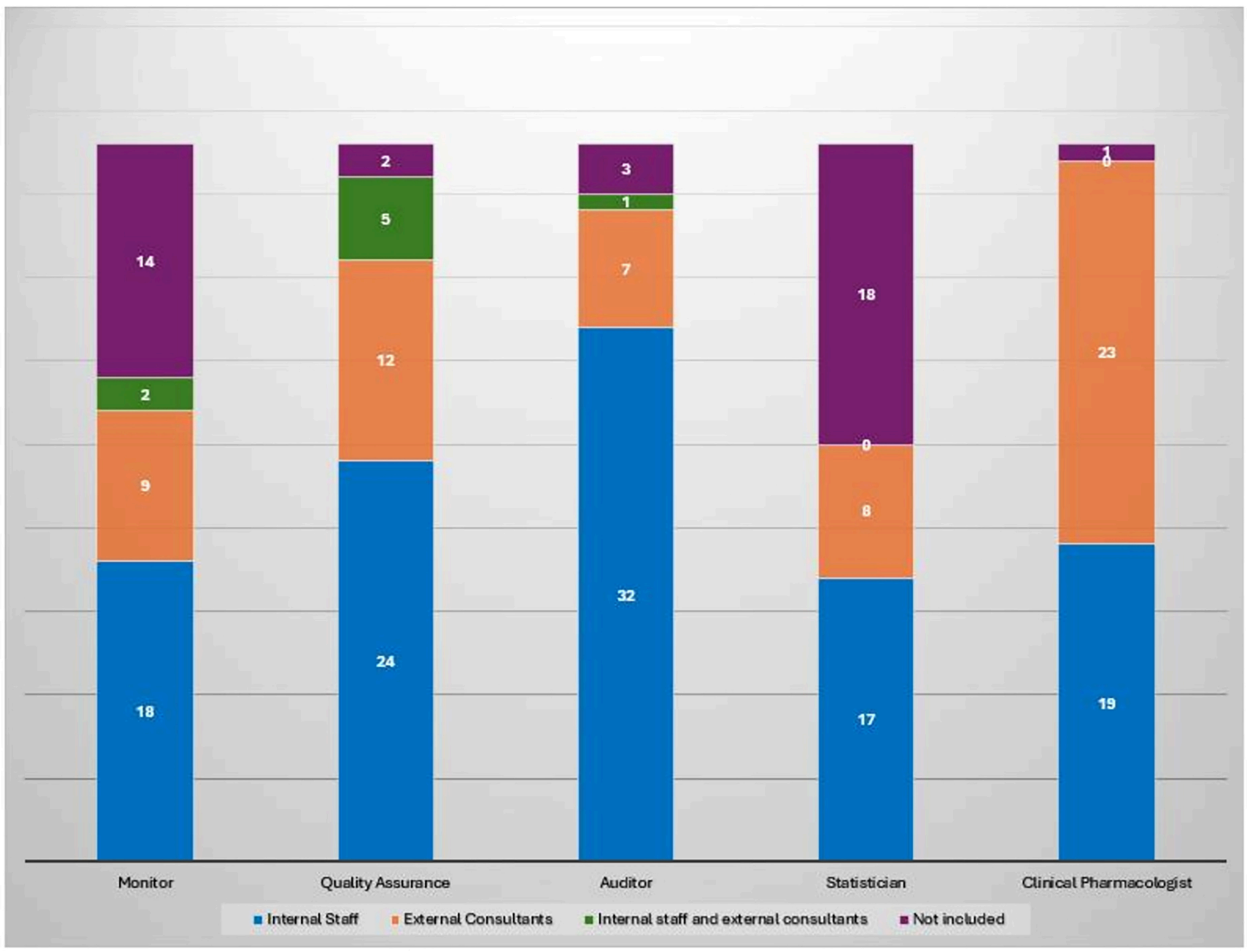
Composition of CTQT roles by type of engagement across 43 institutions with an established or in-progress CTQT. Each bar represents the total number of institutions (*n* = 43) per role, subdivided by engagement type.

**FIGURE 3:**
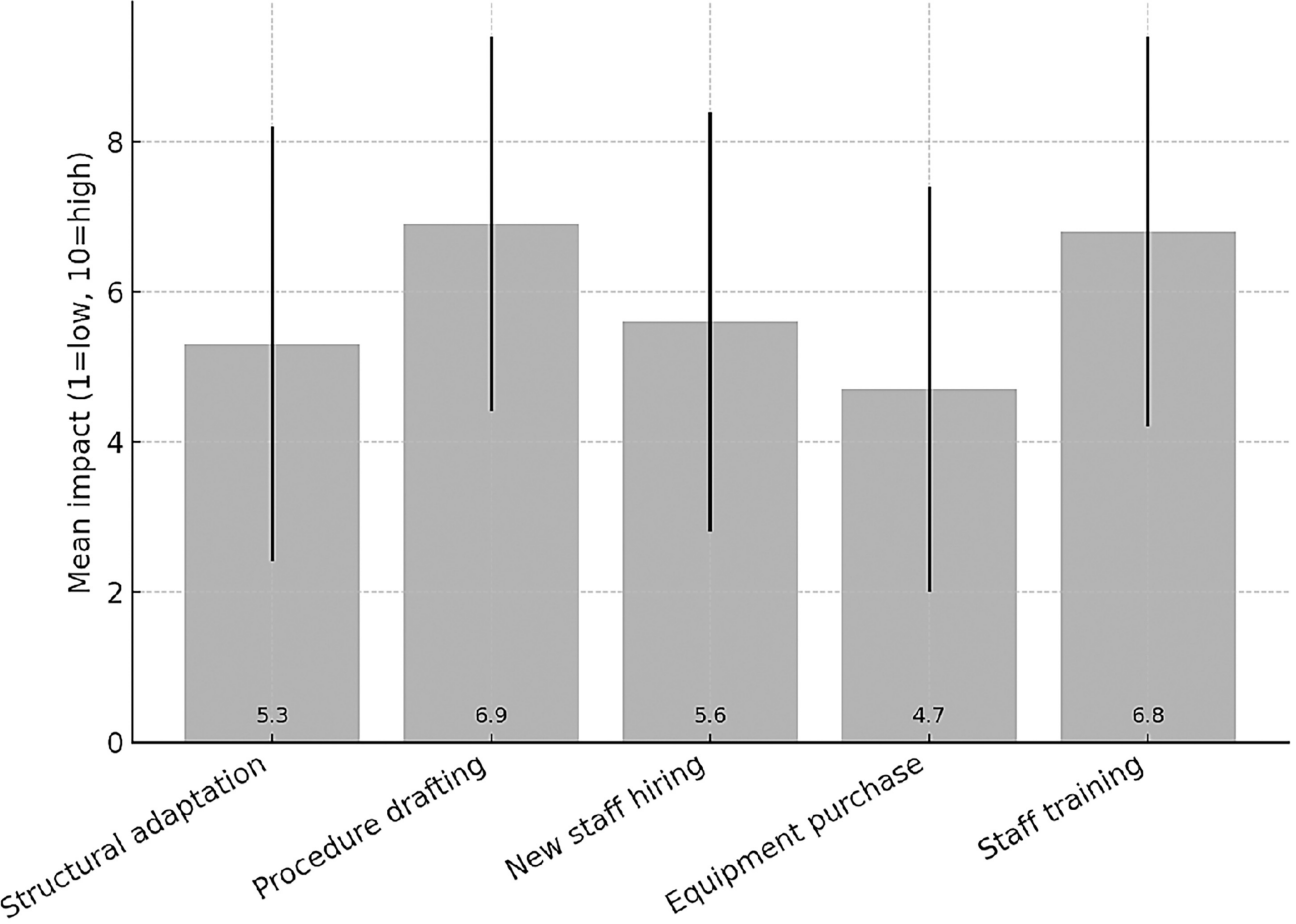
Impact of different areas on the self-certification process.

Among the 62 responding institutions, 39 (62.9%) reported having institutionally established a Clinical Trial Quality Team (CTQT), while 4 (6.5%) indicated that it was under constitution; 19 institutions (30.6%) reported not having a CTQT. Analysis of the 43 institutions with an established or in-progress CTQT showed a heterogeneous composition (Figure 2). The Clinical Pharmacologist was present in 97.7% (*n* = 42/43) of CTQTs, predominantly as external consultants (53.5%, *n* = 23/43) or internal staff member (44.2%, *n* = 19/43). The Quality Assurance specialist was present in 95.3% (*n* = 41/43), mostly as internal staff (55.8%). The Monitor was present in two-thirds of CTQTs (67.4%, *n* = 29/43), mainly as internal staff (41.9%, *n* = 18/43), whereas the Auditor was most often contracted as an external consultant (74.4%, *n* = 32/43). Statisticians were less frequently represented, being absent in 41.9% (*n* = 18/43) of CTQTs and, when present, most commonly internal (39.5%, *n* = 17/43). Participants were invited to rate (scale 1–10) the impact of various organizational and regulatory components of the AIFA Determination 809/2015 on their self-certification pathway (Figure 3). Procedure drafting was rated as the most burdensome aspect (mean 6.9, SD 2.5), closely followed by staff training (mean 6.8, SD 2.6). A moderate perceived burden was associated with new staff recruiting (mean 5.6) and structural adaptation (mean 5.3), while equipment purchase was considered least impactful (mean 4.7).

**FIGURE 4:**
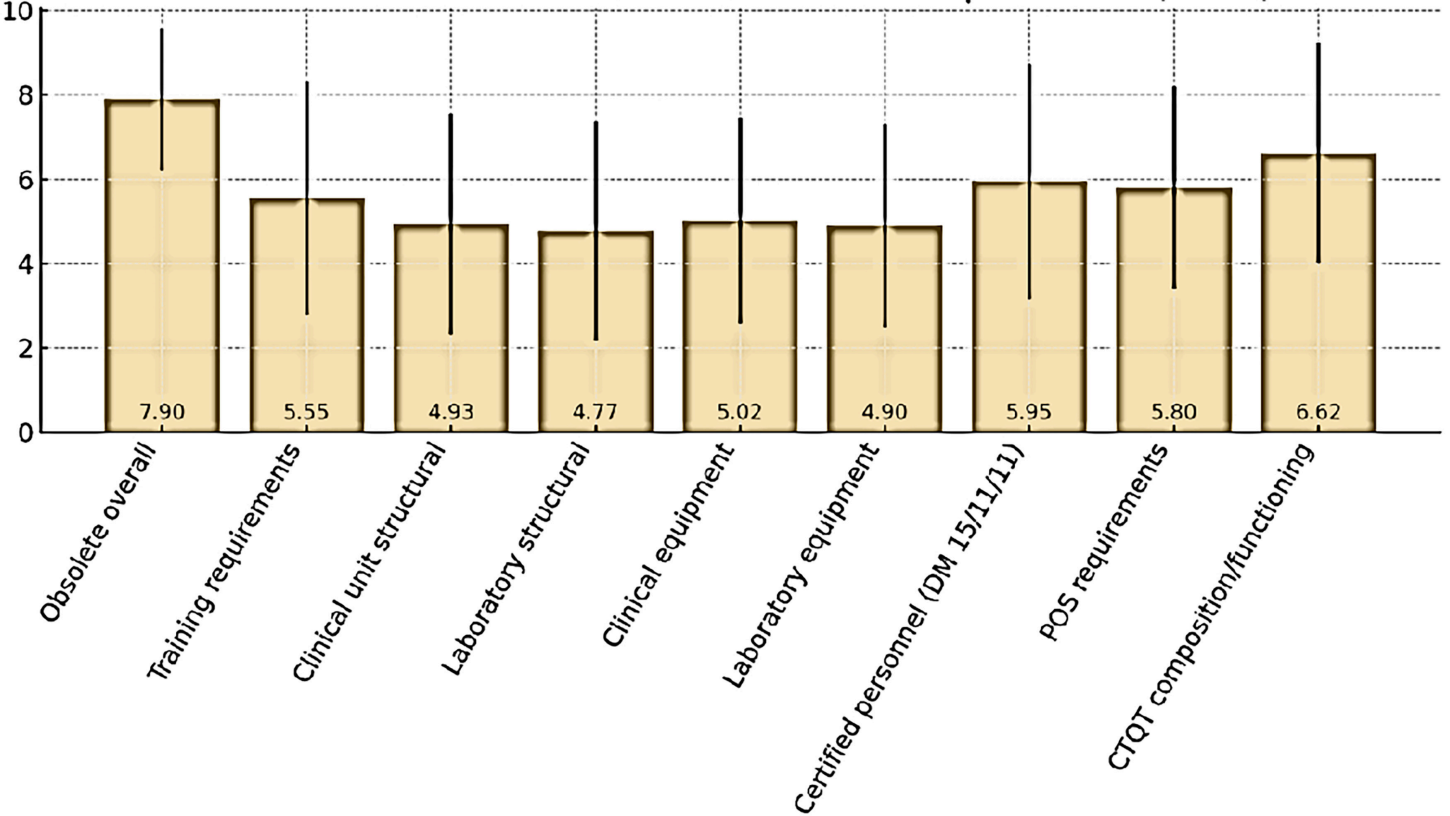
Perceived obsolescence (mean± SD) of Determination requirements.

Respondents also expressed their views on the perceived obsolescence of the current Determination and its selected regulatory requirements (Figure 4). Overall, the Determination was considered largely outdated, with a mean score of 7.90 ± 1.68 (median 8). The domains perceived as most obsolete were CTQT composition and functioning (6.62 ± 2.62, median 7) and certified personnel requirements under DM 15/11/11 (5.95 ± 2.78, median 6.5). Moderate obsolescence was attributed to SOP requirements (5.80 ± 2.40, median 6) and training requirements (5.55 ± 2.78, median 6). By contrast, lower ratings were assigned to structural requirements for clinical unit (4.93 ± 2.62, median 5) and laboratory (4.77 ± 2.59, median 5), as well as to equipment requirements for clinical units (5.02 ± 2.44, median 5) and laboratory (4.90 ± 2.41, median 5).

Finally, respondents were asked to rate the feasibility of extending the Determination requirements to later-phase studies (Table 7). The mean feasibility score was 6.21 ± 2.23 (median 6.5), with responses ranging from 1 to 10. Half of the respondents (*n* = 31/62, 50%) rated feasibility as high (scores 7–10), while 37.1% (*n* = 23/62) gave intermediate ratings (4–6) and 12.9% (*n* = 8/62) perceived low feasibility (1–3).

**TABLE 7:** Feasibility of extending determination requirements to other study phases.

Extending determination requirements to other study phases	N	Mean	Median	SD
	62	6.18	6.50	2.16
**Score**	**Count**		**Percentage**	
1	2		3.2	
2	2		3.2	
3	4		6.5	
4	5		8.1	
5	8		12.9	
6	10		16.1	
7	13		21.0	
8	11		17.7	
9	4		6.5	
10	3		4.8	

Ratings were provided on a 10-point Likert scale (1= not feasible at all, 10 = highly feasible). SD, standard deviation.

## Discussion

### Overview of main findings

This survey provides a comprehensive overview of the operational, regulatory and organizational characteristics of Italian phase I units in the post-implementation phase of AIFA Determination 809/2015. The results highlight both the achievements and the persistent challenges faced by centers engaged in early phase clinical research, confirming the central role of this regulation in shaping the national landscape and supporting the development of innovative therapies.

### Operational and professional landscape

The predominance of operational roles such as Data Managers and Clinical Research Coordinators (50.0%) among respondents reflects the increasingly complex logistical and documentation demands of phase I trials. These professionals play a critical role in study management, data integrity and regulatory compliance, particularly given the stringent requirements associated with first-in-human (FIH) and dose-escalation studies. The substantial representation of Quality Assurance (QA) officers (29.0%) further underscores the centrality of quality management systems (QMS) in current practice. This trend is consistent with international regulatory expectations, including those outlined by the International Council for Harmonization (ICH E6 R3) guidelines, which emphasize quality by design and proactive risk management.

Conversely, clinical roles such as investigators and research nurses were less represented. This may partly reflect both survey response bias and the increasing reliance on multidisciplinary operational teams, a trend in line with global practice where operational and regulatory expertise are considered as essential as clinical expertise for trial success.

### Institutional characteristics

The medical focus of the participating centers was highly heterogeneous. Oncology and hematology, either alone or combined, accounted for a significant proportion of phase I activity, consistent with the global predominance of oncology in early phase drug development pipelines. Units open to all therapeutic areas suggests diversification strategies to attract a broader range of sponsors, while the inclusion of niche areas such as pediatrics, transplantation and gene therapy remain limited due to specialized infrastructures needs.

Most respondents reported involvement in both industry-sponsored and non-profit trials (67.7%), reflecting a dual mission of commercial and academic research. However, the limited number of centers conducting exclusively non-profit studies (3.2%) underlines the resource and infrastructural challenges that constrain independent academic initiatives. Procedure drafting and staff training as the most burdensome aspects of the self-certification process. These findings suggest that, while the Determination aims to raise standards, it may also inadvertently have introduced operational barriers, particularly for smaller or less resourced centers.

### Regulatory compliance, inspections, and sustainability

The widespread adoption of self-certification under AIFA Determination 809/2015 demonstrates a strong commitment to regulatory compliance across Italian phase I centers. The Determination introduced a structured and standardized approach to certifying clinical units and laboratories involved in early phase research, aiming to harmonize quality standards nationally. The fact that 71.0% of respondents reported dual certification (clinical unit and laboratory) suggests broad institutional engagement with the regulation.

However, certification timing varied widely from 2016 to 2024 reflecting differences in readiness, resource availability and interpretation of the regulatory requirements. The reported preparatory phase duration, ranging from 1 to 36 months (12-month median), underscores the substantial organizational effort required. These timeframes likely reflect the need for infrastructural adaptation, personnel training, SOP development and internal audits to certification, confirming the significant administrative burden during regulatory transitions.

Inspection data provide additional insight into compliance challenges. More than half of the inspected units reported major or critical deviations, highlighting the difficulty of maintaining continuous adherence to regulatory requirements. Voluntary suspension of certification (reported by 21.0% of centers) often reflected either critical non-conformities during internal quality checks or the inability to sustain compliance over time, revealing the fragility of long-term regulatory sustainability—particularly in resource-constrained environments.

### Clinical trial quality teams

The establishment of Clinical Trial Quality Teams (CTQTs) appears well integrated into institutional practice. Most centers have adopted a multidisciplinary approach, with the inclusion of principal investigators, CRCs, data managers, pharmacists and QA officers. However, the underrepresentation of roles such as regulatory affairs specialists (30.6%), administrative staff (17.7%) and IT/data protection officers (16.1%) suggests variability on how CTQTs are structured, with possible implications for both regulatory compliance and operational efficiency. Broader team composition could enhance resilience especially given GDPR and complex submission requirements.

Resource allocation further emerged as a critical issue: while QA officers and clinical pharmacologists are consistently integrated into institutional staff, other profiles such as certified monitors, auditors and statisticians are often outsourced or absent, pointing to uneven institutional investment in permanent quality oversight infrastructure. The nearly universal presence of clinical pharmacologists (96.8%) reflects their essential role in phase I research. This reliance on external consultants may reduce continuity and weaken institutional knowledge retention, underscoring uneven investment in permanent quality oversight infrastructure.

### Perceived burdens and comparative perspective

Respondents perceived procedure drafting and staff training as the most burdensome aspects of the self-certification process. These findings suggest that, while the Determination aims to raise standards, it may also inadvertently have introduced operational barriers, particularly for smaller or less resourced centers.

When viewed in a broader European context, different regulatory philosophies become apparent. The Italian model enforces a mandatory self-certification framework that ensures continuous institutional accountability but demands sustained resources. In contrast, other countries adopt voluntary accreditation or inspection-based models that combine flexibility with oversight. The British system, based on periodic renewal and structured review, promotes continuous improvement, whereas the Italian framework emphasizes long-term institutional responsibility. These contrasting approaches illustrate the balance between flexibility and control, suggesting that the introduction of periodic review mechanisms could enhance sustainability while preserving Italy’s rigorous safety and quality standards.

### Policy implications and future directions

The survey indicates a strong consensus in favor of revising and updating the regulatory framework. While most participants support revision of the AIFA Determination 809/2015, they appear to advocate for targeted refinements rather than wholesale reform. Domains perceived as increasingly obsolete (such as CTQT composition or personnel certification requirements) could benefit from modernization, while maintaining safeguards that ensure participant protection and data reliability.

AIFA Determination 809/2015 has significantly contributed to the standardization and quality of Italian phase I research, but selective regulatory refinement is needed to preserve core quality principles while reducing unnecessary administrative burdens. Greater flexibility in staffing models, updated monitoring approaches and alignment with international risk-based quality management could strengthen sustainability.

Finally, supporting non-profit early phase research could help balance commercial and academic priorities, ensuring scientific innovation alongside market-driven development. Overall, tailoring the regulatory framework to balance rigor with feasibility could promote sustainable growth of early phase research while maintaining high standards of patient safety and data integrity.

## Conclusion

This national survey provides a unique insight into the current landscape of Italian phase I units nearly a decade after the introduction of AIFA Determination 809/2015. The findings confirm that the regulation has fostered a culture of quality and safety, but they also highlight persistent operational and structural challenges, particularly for academic and public institutions.

While the widespread adoption of self-certification and Clinical Trial Quality Teams (CTQT) demonstrates institutional commitment, the variability in certification timelines, the frequent outsourcing of key professional roles and the relatively high rate of deviations and voluntary suspensions point to areas where sustainability remains fragile. Moreover, respondents consistently emphasized the regulatory and procedural burden associated with compliance, underscoring the need for a more proportionate and flexible framework.

Overall, the results suggest that a selective revision of AIFA Determination 809/2015 is both timely and necessary. Targeted updates—particularly regarding CTQT composition, personnel certification and procedural requirements—could help preserve high safety and quality standards while enhancing feasibility, operational efficiency and competitiveness of Italian phase I research. Strengthening the balance between regulatory rigor and practical sustainability will be essential to support innovation, maintain alignment with international best practices and promote greater involvement of academic and non-profit sectors in early phase trials.

## Data Availability

The original contributions presented in this study are included in the article/Supplementary material, further inquiries can be directed to the corresponding author.
