# Supplementary material for: The future of Italian phase I trials regulation: lessons from a nationwide survey
Source: Front Med (Lausanne). 2025 Nov 12;12:1709108. doi: 10.3389/fmed.2025.1709108 (PMC12647064; doi:10.3389/fmed.2025.1709108)
Supplement: Supplementary file 1 [file Table_1.docx]

## Supplementary Material – Appendix 1

### Full Survey Questionnaire (English Translation)

#### Section 1: Respondent and Institutional Information

1. What is your professional role?
   - Clinical Research Coordinator / Data Manager
   - Principal Investigator
   - Pharmacist
   - Quality Assurance (QA) / Auditor
   - Laboratory Staff
   - Hospital Administrator / Director
   - Other (please specify)
2. What type of unit do you represent?
   - Oncology
   - Hematology
   - Pediatric Onco-Hematology
   - Other (please specify)
3. Does your center have a dedicated Phase I unit?
   - Yes
   - No
4. In what year did your institution obtain self-certification according to AIFA Determination 809/2015?
   - [Dropdown list: 2015–2024]
   - Not yet certified
   - In progress
5. Is your center certified for non-profit Phase I trials?
   - Yes
   - No
   - In progress

#### Section 2: Operational Activity

1. How many Phase I studies has your center conducted in the past 12 months?
   - 0
   - 1–5
   - 6–10
   - 10
2. Are you involved in studies with:
   - Profit sponsors
   - Non-profit sponsors
   - Both
   - Healthy volunteers
   - Patients
   - All of the above

#### Section 3: Personnel and Quality Systems

1. Which certified professionals are present at your site?
   (Select one option per row: Internal / External Consultant / Both / Not Available)

| **Role** | **Internal** | **Consultant** | **Both** | **Not Present** |
| --- | --- | --- | --- | --- |
| Certified Monitor | ☐ | ☐ | ☐ | ☐ |
| Certified QA Specialist | ☐ | ☐ | ☐ | ☐ |
| Certified Auditor | ☐ | ☐ | ☐ | ☐ |
| Certified Statistician | ☐ | ☐ | ☐ | ☐ |
| Clinical Pharmacologist | ☐ | ☐ | ☐ | ☐ |

1. Is a Clinical Trial Quality Team (CTQT) active in your institution?
   - Yes
   - No
   - In progress
2. Do you have Standard Operating Procedures (SOPs) specific to Phase I research?

- Yes, fully implemented
- In progress
- No, but planned
- No

1. How is GCP (Good Clinical Practice) training provided to your staff?

- Internal courses
- External training
- Both
- Not provided

#### Section 4: Perceptions and Regulatory Evaluation

1. On a scale from 1 (not impactful) to 10 (extremely impactful), how much did each of the following aspects affect your institution’s ability to obtain Phase I self-certification?

| **Factor** | **1–10** |
| --- | --- |
| Structural adjustments |  |
| SOP development |  |
| Hiring specialized personnel |  |
| Purchase of new equipment |  |
| Staff training |  |

1. In your opinion, to what extent is it feasible to extend the requirements of AIFA Determination 809/2015 to later-phase trials (e.g., Phase II–III)?

- [Likert scale: 1 = Not feasible at all, 10 = Completely feasible]

1. How much do you believe that Determination 809/2015 is outdated and needs revision?

- [Likert scale: 1 = Not at all, 10 = Definitely]

1. Which aspects of the regulation should be updated?
   (Score from 1 = still adequate to 10 = urgently needs revision)

| **Regulatory Area** | **1–10** |
| --- | --- |
| Personnel training requirements |  |
| Structural requirements of clinical units |  |
| Laboratory infrastructure requirements |  |
| Required equipment for clinical units |  |
| Required equipment for laboratories |  |
| Certified staff roles under Ministerial Decree |  |
| CTQT structure and functioning |  |
| SOP-related requirements |  |

1. What are the most critical or limiting aspects of the Determination in your daily experience?
   (Open text)
2. Do you think the current Determination restricts Italy’s competitiveness in attracting international sponsors?

- Yes
- No
- Not sure

1. Would you support a proposal to implement differentiated certification paths (e.g., modular or tiered system)?

- Yes
- No
- I would need more information

1. Additional comments or suggestions for AIFA or the national regulatory framework:
   (Open text)
